# Natural Killer Cells and Anti-Cancer Therapies: Reciprocal Effects on Immune Function and Therapeutic Response

**DOI:** 10.3390/cancers13040711

**Published:** 2021-02-09

**Authors:** Elisa C. Toffoli, Abdolkarim Sheikhi, Yannick D. Höppner, Pita de Kok, Mahsa Yazdanpanah-Samani, Jan Spanholtz, Henk M. W. Verheul, Hans J. van der Vliet, Tanja D. de Gruijl

**Affiliations:** 1 Cancer Center Amsterdam, Department of Medical Oncology, Amsterdam UMC, Vrije Universiteit Amsterdam, De Boelelaan 1117, 1081 HV Amsterdam, The Netherlands; e.toffoli@amsterdamumc.nl (E.C.T.); sheikhi@queensu.ca (A.S.); yannick.hoeppner@bnitm.de (Y.D.H.); pitadk@outlook.com (P.d.K.); jj.vandervliet@amsterdamumc.nl (H.J.v.d.V.); 2 Department of Immunology, School of Medicine, Dezful University of Medical Sciences, Dezful 64616-43993, Iran; 3 Department of Medical Biotechnology, School of Advanced Medical Sciences and Technologies, Shiraz University of Medical Sciences, Shiraz 71348-45794, Iran; myazdanpanah@sums.ac.ir; 4 Glycostem, Kloosterstraat 9, 5349 AB Oss, The Netherlands; jan@glycostem.com; 5 Department of Medical Oncology, Radboud Institute for Health Sciences, Radboud University Medical Center, Geert Grooteplein Zuid 10, 6525 GA Nijmegen, The Netherlands; Henk.Verheul@radboudumc.nl; 6 Lava Therapeutics, Yalelaan 60, 3584 CM Utrecht, The Netherlands

**Keywords:** NK cells, radiotherapy, local ablation therapies, checkpoint inhibitors, chemotherapy, protein kinase inhibitors, oncolytic virus, cancer, anti-cancer therapies

## Abstract

**Simple Summary:**

Natural Killer (NK) cells are innate lymphocytes that play an important role in the immune response against cancer. Their activity is controlled by a balance of inhibitory and activating receptors, which in cancer can be skewed to favor their suppression in support of immune escape. It is therefore imperative to find ways to optimize their antitumor functionality. In this review, we explore and discuss how their activity influences, or even mediates, the efficacy of various anti-cancer therapies and, vice versa, how their activity can be affected by these therapies. Knowledge of the mechanisms underlying these observations could provide rationales for combining anti-cancer treatments with strategies enhancing NK cell function in order to improve their therapeutic efficacy.

**Abstract:**

Natural Killer (NK) cells are innate immune cells with the unique ability to recognize and kill virus-infected and cancer cells without prior immune sensitization. Due to their expression of the Fc receptor CD16, effector NK cells can kill tumor cells through antibody-dependent cytotoxicity, making them relevant players in antibody-based cancer therapies. The role of NK cells in other approved and experimental anti-cancer therapies is more elusive. Here, we review the possible role of NK cells in the efficacy of various anti-tumor therapies, including radiotherapy, chemotherapy, and immunotherapy, as well as the impact of these therapies on NK cell function.

## 1. Introduction

**Figure 1:**
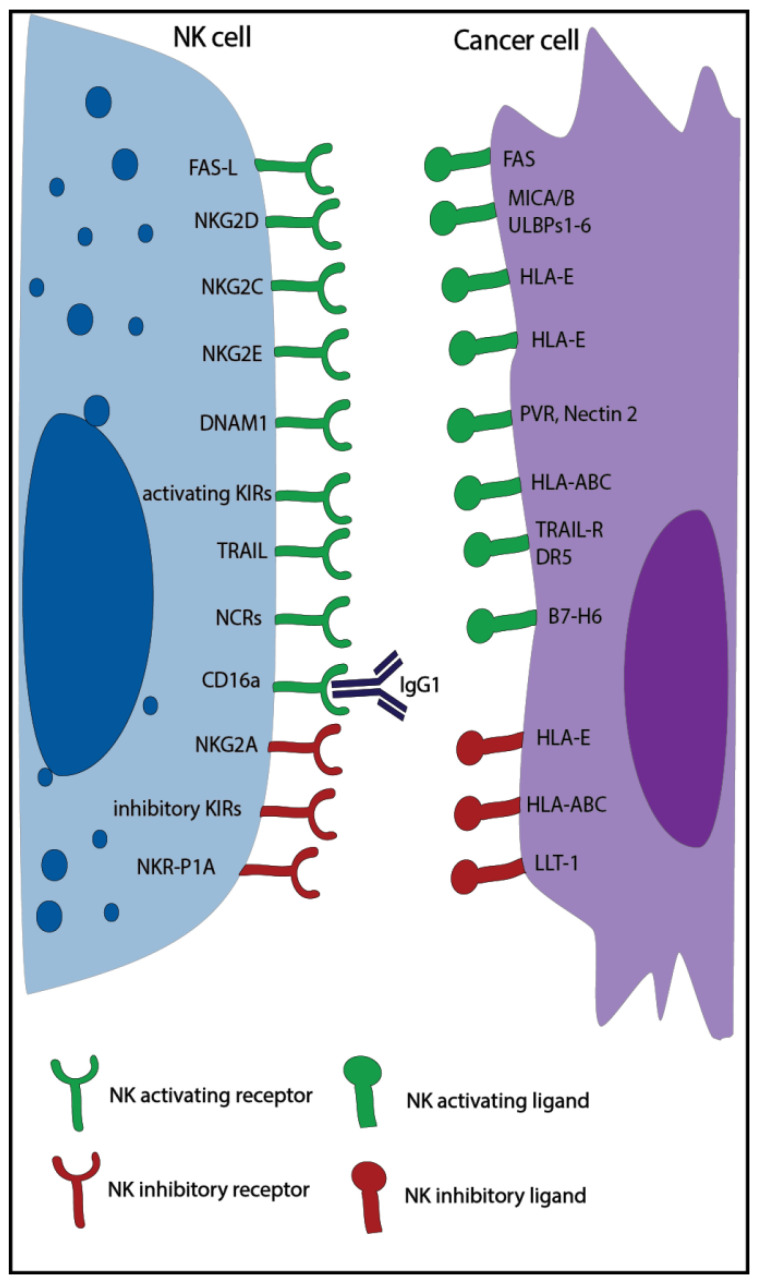
Overview of major natural killer (NK) cell activating and inhibitory receptors and their corresponding ligands expressed on tumor cells. KIR: killer cell immunoglobulin-like receptors; NKG: natural killer receptor; TRAIL: tumor necrosis factor-related apoptosis-inducing ligand; NCR: natural cytotoxicity receptors; HLA: human leukocyte antigen; MICA: MHC class I polypeptide–related sequence; PVR: poliovirus receptor; DR5: death receptor 5; DNAM1: DNAX Accessory Molecule-1, ULBP1-6: UL16 binding protein 1-6, LLT-1: lectin-like Transcript-1.

Natural killer (NK) cells are large granular lymphocytes, part of the innate immune system, and are characterized by the expression of CD56, the absence of CD3 [1], and the ability to kill virus-infected and tumor cells without prior immune sensitization [2]. Classically, NK cells can be divided into two main subsets with distinct properties: the CD56^dim^ effector NK cells with high cytotoxic capacity and the CD56^bright^ NK cells, which exert mainly a regulatory function [3]. NK cell activity is controlled by a balance of inhibitory and activating receptors (Figure 1) [1]. Killer cell immunoglobulin-like receptors (KIR) and the natural killer receptor (NKG) 2A are examples of inhibitory receptors that are important to suppress non-specific cytotoxic activity and killing of healthy cells. They bind to multiple human leukocyte antigens (HLA), which can be downregulated by tumor and virus-infected cells to escape T cell recognition leading to increased NK cell recognition. In addition, NK cells can express other inhibitory receptors recognizing non-MHC molecules, such as the Lectin-like Transcript-1 (LLT-1), which binds to NKR-P1A. The activating receptors, such as natural killer group 2 member (NKG2)D/C/E, and natural cytotoxicity receptors (NCRs), such as NKp30, NKp46, and NKp44, recognize specific ligands, like MHC class I polypeptide–related sequence A/B (MICA/B), UL16 binding protein 1-6 (ULBP1-6), and heparan sulfate proteoglycan (HSPG), that are overexpressed by infected and malignant cells [1,2]. When the balance of these receptors is skewed towards activation, either due to a lack of inhibitory signals or the dominance of activating signals, NK cells are triggered to release cytotoxic granules and cytotoxic effector proteins like granzymes and perforin in order to kill the target cell [4,5]. NK cells can also induce apoptosis via Fas ligand and tumor necrosis factor (TNF)–related apoptosis-inducing ligand (TRAIL), which bind to the Fas receptor and the death receptor 5 (DR5) expressed on target cells [4]. Moreover, thanks to the expression of CD16 (Fc receptor: FcRγIII), NK cells can also kill through antibody-dependent cell-mediated cytotoxicity (ADCC) [6].

Cancer is a leading cause of death worldwide, and the number of new patients diagnosed with cancer is still rising globally [7]. In Europe, 20% of deaths are caused by cancer [8]. Although multiple therapeutic approaches are currently available, cancer remains a clinical challenge, and therefore new insights into this disease are necessary. NK cells are known to play a pivotal role in cancer. Patients with higher NK cell activity were found to have a better prognosis [9,10], and thanks to their ability to kill circulating cancer stem cells, which have high metastatic potential, NK cells play an essential role in the prevention of metastasis [11]. While the role of NK cells in monoclonal antibody-based therapies is well established [12], less is known on the function that NK cells have in other anti-cancer therapies. This review will focus on the effects that various anti-cancer therapies have on NK cells and the possible role that NK cells play in these therapies. An overview of clinical observations is presented in Supplementary Table S1.

## 2. Radiotherapy

**Figure 2:**
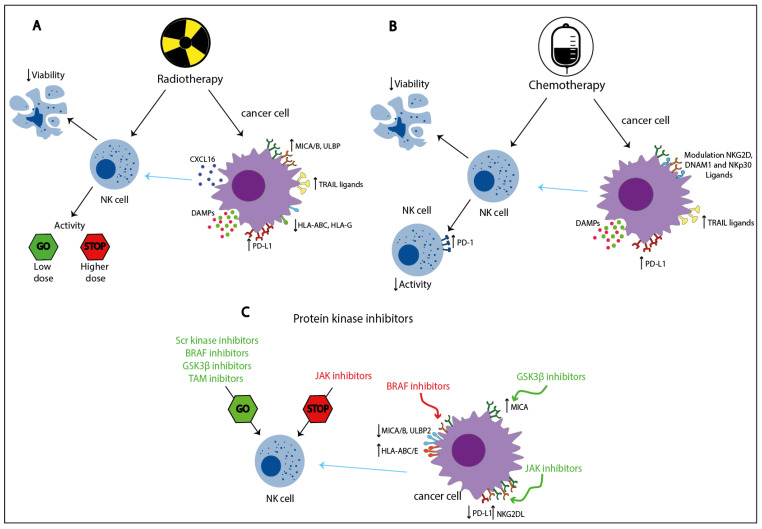
Direct and indirect effects of radiotherapy, chemotherapy, and protein kinase inhibitors on NK cell activity. Radiotherapy (**A**) and chemotherapy (**B**) cause cell damage, often leading to NK cell impairment, whereas protein kinase inhibitors (**C**) target specific signaling pathways resulting in either increased or decreased NK cell activity depending on the pathway involved. These treatments can also induce the modulation of various NK cell ligands on tumor cells and the release of damage-associated molecular patterns (DAMPs), indirectly affecting NK cell functions. Red arrows: inhibitory effects; green arrows: stimulatory effects; blue arrows: indirect effects, modulating the tumor cell’s susceptibility to NK-mediated cytolysis. ULBP1-6: UL16 binding protein 1-6; MICA: MHC class I polypeptide–related sequence; TRAIL: tumor necrosis factor-related apoptosis-inducing ligand; HLA: human leukocyte antigen; PD-(L)1: programmed cell death protein (ligand) 1, NKG: natural killer receptor; NCR: natural cytotoxicity receptors; BRAF: B-rapidly accelerated fibrosarcoma; GSK-3β: Glycogen synthase kinase-3β; JAK: Janus kinase.

Radiotherapy is an anti-cancer strategy based on the administration of ionizing radiation, which induces DNA damage and cell death, and that is currently included in more than 50% of all anti-cancer treatments [13,14]. Radiotherapy affects NK cells both directly and indirectly (Figure 2).

The viability of ex vivo irradiated NK cells from healthy donors was shown to be reduced, and this decrease was directly correlated with a higher single radiation dose and the length of the post-irradiation measurement interval [15,16,17]. For instance, the mean percentage of dead NK cells after the administration of 1 Gy ranged from 1.3% to 20.7% after 2 and 42 h, respectively, whereas at the same time points, a dose of 10 Gy induced the death of 2.7% to 67.5% NK cells [15]. At low radiation doses (<0.1 Gy), *ex vivo* irradiated NK cells from healthy donors actually demonstrated higher levels of cytotoxicity compared to non-irradiated NK cells. Moreover, higher expression of TNFα and interferon-γ (IFNγ) was observed. Interestingly, the addition of a specific P38 inhibitor hampered the positive effect of low dose radiation on NK cell cytotoxicity, suggesting that the p38-mitogen-activated protein kinase (MAPK) pathway might mediate this effect [18]. In another study, occasionally higher NK cell cytotoxicity was found when ex vivo NK cells from healthy donors were irradiated with a single dose between 1–10 Gy compared to non-irradiated cells [15]. In addition, the administration of a total dose of 10 Gy in two fractions was observed to enhance *ex vivo* healthy donor NK cell cytotoxicity compared to the non-fractionated dose [17]. In contrast, a reduction in cytotoxicity was reported when ex vivo isolated NK cells from healthy donors were treated with higher radiation doses (>20 Gy) [15,17].

Multiple studies focusing on patients with cancer undergoing radiotherapy also unveiled reductions in the absolute number of various peripheral blood (PB) lymphocyte subsets, including NK cells [19,20,21,22,23,24,25], and impaired NK cell activity compared to pre-treatment levels [26,27], suggesting that radiotherapy directly decreases both NK cell viability and function in a dose-dependent manner. 

The indirect effects of radiotherapy on NK cells can be divided into three categories: the modulation of activating and inhibitory NK ligands, the release of damage-associated molecular patterns (DAMPs), and the enhancement of NK cell migration to the tumor. Upon radiotherapy, many cell types, including tumor cells, modulate the expression of NK cell ligands with a crucial impact on the sensitization to NK cell responses. Cancer cells from various solid tumor types were discovered to upregulate MICA/B and ULPB1–3 [28,29,30,31], whereas they downregulated the KIR2D ligands HLA-ABC and HLA-G [32,33,34,35], suggesting a higher sensitivity to NK cell-mediated cytotoxicity. Moreover, multiple irradiated cancer cell lines showed an increased expression of the intracellular adhesion molecule 1 (ICAM1), which was described to enhance NK cell-mediated killing by increasing cell-to-cell adhesion, and the Fas receptor, possibly indicating higher susceptibility to NK cell-mediated apoptosis [32,33,36]. Of note, also cancer stem cells (CSC), which represent a small radio-resistant population, were found not only to upregulate the Fas receptor in an irradiation dose-dependent manner but also to upregulate MICA/B, suggesting higher sensitization to NK cell killing [37]. On the other hand, other irradiated cancer cell lines demonstrated to be more resistant to NK cell cytotoxicity by the downregulation of MICA/B, ULPB 1-3, or the upregulation of HLA-ABC [33,38]. It is important to note that different tumor cell lines were used to analyze these effects and that the discrepancies in the responses could be due to cell line specific properties. Indeed, a study analyzing expression levels of various proteins related to NK cell sensitivity (e.g., of Fas, HLA-ABC) on human colon, lung, and prostate cancer cell lines upon irradiation found heterogeneous responses [33]. Moreover, variation in the expression of NKG2D ligands (NKG2D-L; e.g., MICA/B, ULBP1-3) might be due to the upregulation of matrix-bound metalloproteinases (MMPs) by cancer cells, which can shed NKG2D-L from the tumor cell surface leading to decreased membrane expression, consequently reducing NK cell recognition and activation [31]. 

Radiotherapy can also induce the release of DAMPs by tumor cells, such as heat shock proteins (Hsp), which are a family of stress-inducible factors with anti-apoptotic function regularly expressed by tumor cells [39]. Higher levels of Hsp70 are produced in response to cellular stress, which can be caused by radiotherapy [40,41]. In addition to the intracellular anti-apoptotic function, the release of Hps70, or its expression on the cell surface, can function as a DAMP triggering anti-tumor immune responses. In particular, membrane-bound Hsp70 (mHsp70) can elicit NK cell activation and tumor cell killing through binding to NKG2A/C/E and the co-receptor CD94 [42,43]. However, the expression of HLA-E by tumor cells can hinder this mechanism, significantly reducing Hsp70-dependent activation [44]. Radiotherapy and genotoxic stress can also induce the release of other DAMPs such as adenosine triphosphate (ATP), which can be bound and processed into adenosine (ADO) by multiple cells in the tumor microenvironment (TME) (e.g., tumor cells, regulatory T cells (Treg), and CD8^+^ T cells) through their expression of CD39 and/or CD73. ADO is a highly immunosuppressive factor that impairs the function of multiple immune cells through the binding with its receptor, which is also expressed by NK cells [45,46].

Finally, radiotherapy enhanced NK cell migration to tumor cells in vitro. Multiple irradiated breast cancer cell lines were shown to increase their in vitro production of CXCL16, a CXCR6 ligand, leading to higher NK cell trans-well migration [47]. In conclusion, radiotherapy directly impaired NK cell viability and activity in a dose-dependent manner while modulating tumor cell sensitivity to NK cell-mediated cytotoxicity and the TME, potentially both promoting and impairing NK cell function, depending on dose and tumor heterogeneity, suggesting that combining radiotherapy with strategies to maintain NK cell viability and activity could be beneficial. 

## 3. Local Ablation Therapies

Local ablation therapies are minimally invasive therapeutic approaches, whereby tumor cell death is caused by locally applied heat, cold, ultrasound, microwaves, irreversible electroporation, high-frequency electromagnetic currents, or chemicals. Here, we will focus on the effects of thermal ablation therapies on NK cells because, to our knowledge, the effects of other approaches on NK cells have not been investigated.

### 3.1. Radiofrequency Ablation

Radiofrequency ablation (RFA) is a technique based on alternating electromagnetic currents, which, by inducing ionic agitation, create local heat and, consequently, induce tumor necrosis [48,49]. RFA was found to positively affect NK cell frequency and activation. Multiple tumor-bearing animal models (i.e., mouse, rabbit, and rat) exhibited higher levels of peripheral blood (pb)NK cells after treatment with RFA [50,51]. Moreover, tumor-bearing rats treated with RFA had higher levels of intratumoral NK cells compared to untreated rats [52]. The effects of RFA on NK cell activation were studied in a rabbit model where higher *ex vivo* production of IFNγ and TNFα by NK cells was found after treatment as well as higher *ex vivo* cytotoxic activity compared to NK cells from non-treated rabbits, which was dependent on the expression of NKG2D [51]. 

In humans, no differences in pbNK cell percentages were measured after RFA compared to baseline in patients with renal cell carcinoma [53]. In contrast, patients with hepatocellular carcinoma (HCC) showed higher levels of pbNK cells after RFA treatment [54,55]. This increment was described to be mostly due to elevated levels of CD56^dim^ pbNK cells, which were accompanied by increases in the expression of various NK cell-activating receptors (NKG2D, CD16, NKp30, and NKp46), a reduction in the expression of the inhibitory receptor NKG2A and higher *in vitro* NK cell cytotoxicity, ADCC, and IFNγ production compared to baseline, suggesting that RFA enhances NK cell activation. Interestingly, elevated levels of IFNγ release and NK cell cytotoxicity at 4 weeks after RFA were associated with longer disease-free survival [54]. However, in a cohort of 80 patients with HCC, a reduction in the percentage of NKp30^+^ pbNK cells occurred one day after RFA treatment, which normalized after one month. In this study, higher frequency of NKp30^+^ NK cells 1 day after RFA correlated to lower tumor recurrence, whereas a delayed increase of total NK cells and higher percentages of CD56^bright^ NK cells at 1 month after RFA were associated with more tumor recurrence in patients with HCC [56], suggesting that NK cell dynamics play an important role in response to this therapy.

### 3.2. Microwave Ablation Therapy

Microwave ablation therapy (MWA) is a treatment whereby, similarly to RFA, electromagnetic energy is used to increase intratumoral temperature and, consequently, induce tumor necrosis. MWA is different as it uses frequencies ≥ 900 kHz, whereas this is lower for RFA treatments (400–500 kHz) [57]. The effects of MWA on NK cells have been studied in two mouse tumor models where higher levels of NK cells were found in PB as well as infiltrating in MWA-treated tumors. Moreover, MWA was shown to induce NK cell activation, which was dependent on intratumoral macrophage-derived interleukin(IL)-15. In the same study, NK cell depletion, but not CD4^+^ or CD8^+^ T cell depletion, significantly decreased overall survival and metastatic control, suggesting that NK cells played a pivotal role in response to MWA [58].

In humans, higher levels of tumor-infiltrating NK cells were described in patients with HCC treated with MWA. Interestingly, in this study, MWA also triggered (though to a lesser extent) NK cell infiltration in distant (non-MWA treated) lesions suggesting that the treatment could have induced an abscopal effect [59]. The frequency of pbNK cells has also been studied in patients with HCC, but no significant differences were shown before and after treatment [60]. In contrast, MWA-treated patients with early-stage breast cancer exhibited higher levels of pbNK cells compared to untreated controls [58]. NK cell activity has been studied in patients with prostate cancer (PC) treated with MWA, where higher levels of *ex vivo* NK cell cytotoxicity were described compared to baseline levels. Interestingly, MWA-treated patients with severely symptomatic benign prostatic hyperplasia exhibited no increases [61], suggesting that specifically tumor-related factors enhanced NK cell effector function. Finally, in patients with HCC or prostate cancer, higher *in vitro* NK cell cytotoxicity and intratumoral NK cell frequency after MWA treatment have both been correlated to therapeutic response [59,61], suggesting that in humans, NK cells also play an important role in response to this therapy.

### 3.3. High Intensity Focused Ultrasound Ablation

High intensity focused ultrasound ablation (HIFU) is a relatively new hyperthermia technique, whereby ultrasound waves induce the oscillation of water molecules to increase the intratumoral temperature (≥60 °C) and consequently, induce cell damage [48]. HIFU therapy has been shown to be an effective treatment approach in multiple solid cancers (e.g., liver, breast, and pancreatic cancer) [62]. The frequency of pbNK cells after HIFU has been studied in patients with various solid malignancies and patients with uterine fibroids and exhibited no difference compared to pre-treatment levels [63,64]. However, higher levels of pbNK cells, assessed using an enzyme-linked immunosorbent assay, were observed after HIFU treatment in patients with primary liver cancer [65]. NK cell tumor infiltration after HIFU was studied in patients with breast cancer, where higher infiltration of CD57^+^ cells was described after treatment [66]. However, CD57 can also be expressed by, e.g., senescent T cells, making this analysis non-specific for NK cells [67]. 

In conclusion, in animal models, both RFA and MWA seem to positively affect NK cell activity and frequency, both intratumorally and peripherally, whereas more discrepancies can be found in human studies, possibly due to differences in analyzed tumor types. The frequency of NK cells has also been studied after HIFU; however, the impact of this therapy on NK cells is less clear. Finally, NK cells seem to play a positive role in determining treatment response to both MWA and RFA. However, due to the small amount of available data, more studies are needed to further clarify and confirm these results. 

## 4. Checkpoint Inhibitors 

### 4.1. PD-1-PD-L1 Axis

**Figure 3:**
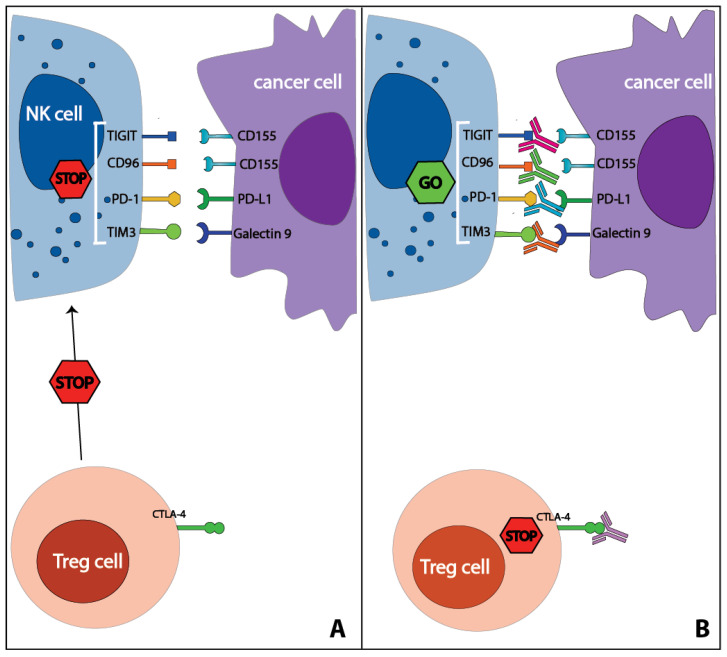
Effects of immune checkpoint inhibitor therapies on NK cells. NK cells can express various immune checkpoints such as PD-1, TIM3, TIGIT, CD96, which, upon binding to their ligands, impair NK cell function (**A**). Antibodies targeting these immune checkpoints were shown to restore NK cell activity (**B**). CTLA-4 is not expressed by human NK cells; however, anti-CTLA-4 therapy positively affects NK cell activity by reducing Treg-mediated suppression. A selection of immune checkpoint ligands is shown in the figure. TIGIT: T cell immunoglobulin and immunoreceptor tyrosine-based inhibitory motif domain; PD-(L)1: programmed cell death protein (ligand) 1; TIM3: T-cell immunoglobulin- and mucin-domain-containing molecule-3; CTLA-4: cytotoxic T-lymphocyte-associated protein 4.

Programmed cell death protein 1 (PD-1) and its ligand (PD-L1) are known for their role in controlling T cell activation, and multiple antibodies targeting this pathway are currently approved for clinical use [68]. Recent studies found that NK cells also play a pivotal part in determining the efficacy of these therapies in both a direct and indirect manner. PD-1^+^ NK cells have been described in patients with multiple cancer types both in PB and, in higher percentages, in the TME [69,70,71,72,73,74]. Multiple studies using both mouse models and human NK cells showed that PD-1^+^ NK cells became impaired upon binding to PD-L1 [70,71,73,74,75], whereas PD-1-PD-L1 blockade at least partially reversed this impairment [68,70,72,73] (Figure 3). Interestingly, it was found that PD-1-PD-L1 blockade could also indirectly influence NK cells by decreasing the expansion of PD-L1-induced Tregs, which could reduce both NK cell function and survival via the release of TGFβ and the seizing of IL-2 [73,76]. In multiple mouse models, NK cells played a key role in the efficacy of PD-1-PD-L1 blockade in both effector T cell-resistant (i.e., MHC-I^lo^) tumors, where the effects of the therapies were NK cell-dependent, and T cell-sensitive tumors (MHC-I^hi^), where the depletion of either CD8^+^ T cells or NK cells led to similar increases in tumor growth suggesting that also in T cell sensitive tumors, NK cells play a role in the efficacy of PD-1-PD-L1 blockade [75]. In keeping with this notion, no response to PD-1 blockade was found after NK cell depletion in mice lacking T cells [72]. Enhanced *in vitro* NK cell-mediated ADCC was observed by applying the checkpoint inhibitor avelumab, an IgG1 monoclonal antibody (mAb) targeting PD-L1 [77,78]. NK cells also play an indirect role in the efficacy of PD-1-PD-L1 blockade. Via the production of chemokines (CCL5, XCL1) and the cytokine fms-related tyrosine kinase 3 ligand (FLT3-L), NK cells can enhance the migration and maintain the survival of type-1 conventional dendritic cells (DC), a rare CD141^+^ DC population, which demonstrated to be particularly efficient in antigen cross-presentation and to be required for an effective anti-PD-1-response [79]. Moreover, high levels of IFNγ, produced also by activated NK cells, induced the expression of PD-L1 on tumor cells [73,76], which could sensitize PD-L1^−^ tumor to PD-1-PD-L1 checkpoint inhibitor therapies.

Other anti-cancer therapies may be more effective when combined with PD-1-PD-L1 blockade, partially as a result of its activating effects on NK cells. For example, cetuximab-activated NK cells were found to express PD-1 and to induce the expression of PD-L1 on tumor cells upon IFNγ release [73]. Similarly, some chemotherapeutic agents induced PD-1 on NK cells, and higher expression levels of PD-L1 on tumor cells were described after exposure to both chemotherapy and radiotherapy, suggesting that combining these therapies with PD-1-PD-L1 blockade might enhance NK cell activity [38,80,81].

In conclusion, PD-1 is an important regulator of NK cell function, and its blockade was shown to enhance NK cell activity leading to increased tumor control. Moreover, NK cells were shown to play a role in response to PD-1-PD-L1 based therapies against tumors both sensitive and resistant to T cell-mediated cytotoxicity.

### 4.2. CTLA-4

Cytotoxic T-lymphocyte-associated protein 4 (CTLA-4) is a T cell immune checkpoint receptor abundantly expressed by Tregs and, upon stimulation, by cytotoxic T cells. Antibodies targeting CTLA-4 are currently used as a therapy against multiple solid tumors [82,83,84]. Limited knowledge is available on the role of NK cells in CTLA-4-based therapies. Although it was shown that mouse NK cells could express membrane CTLA-4 upon stimulation with IL-2 [85], this has not been confirmed in humans [86]. In mice, anti-CTLA-4 was described to positively affect intratumoral NK cell frequency [87,88]. In humans, higher levels of intratumoral NK cells have been correlated with response to anti-CTLA-4 [89,90]. Moreover, in patients with malignant mesothelioma, a perturbation of the PB CD56bright/dim ratio was observed compared to healthy donors, which normalized in favor of the CD56^dim^ effector NK cells after treatment with tremelimumab, an IgG2 anti-CTLA-4 mAb [91]. Interestingly, in patients with melanoma, lower levels of CD56^dim^ NK cells were also found, which increased after treatment with ipilimumab, the first approved anti-CTLA-4 mAb [92]. These data suggest that CTLA-4 blockade influences NK cell dynamics. One factor that could play a role in these observations is the reduction of Tregs upon anti-CTLA-4 therapy, which can reduce Treg-mediated NK cell impairment and thereby increase NK cell activity [93] (Figure 3). A treatment-induced reduction of tumor load might also positively influence the frequency and function of NK cells. Finally, anti-CTLA-4 treatment could also stimulate NK cell ADCC. A recent study showed that in mice, anti-CTLA-4 antibodies can trigger NK cell activation and cytotoxicity against Tregs in an FcR-dependent matter [89]. However, ipilimumab, an IgG1 triggering CD16, induced ADCC against Tregs by monocytes and pro-inflammatory macrophages but not by intratumoral NK cells [93,94,95], whereas tremelimumab was shown to trigger ADCC by classical monocytes expressing CD32a, the main IgG2 receptor [96]. Nevertheless, it was shown that pbNK cells could induce a selective reduction of FOXp3^+^ Treg when TILs, derived from patients with head and neck cancer, were co-cultured with isolated NK cells in the presence of ipilimumab [93]. Furthermore, ipilimumab induced ADCC by pbNK cells from healthy volunteers against CTLA-4^+^ melanoma cells [97]. Of note, no NK cell-mediated ADCC was found against other CTLA-4^+^ T cells [93,97], suggesting that other tumor-specific factors might play a role in the selectivity of this process. In conclusion, anti-CTLA-4 indirectly influenced NK cell activity, possibly through the (relative) reduction of Tregs and tumor load. Moreover, ipilimumab induced ADCC *in vitro* by pbNK but not by intratumoral NK cells, possibly due to differences in functionality.

### 4.3. TIM3

The immunoregulatory protein T-cell immunoglobulin- and mucin-domain-containing molecule-3 (TIM3) is an immune checkpoint receptor originally identified on T cells, which, upon binding with its cognate ligands (e.g., Galactin-9, phosphatidylserine,) inhibits T cell activity [68]. TIM3 has been described on pbNK cells both from healthy donors and, at higher levels, from patients with cancer and on intratumoral NK cells [74,98,99,100,101]. Purified TIM3^+^ NK cells, both from HCC-bearing mice and patients with melanoma, were found to be functionally impaired against tumor cells expressing TIM3 ligands. In both cases, NK cell activity was restored with anti-TIM3 antibodies, which also led to enhanced NK cell proliferation [98,102] (Figure 3). These data were confirmed in two T-cell deficient murine models where anti-TIM3 antibody therapy led to prolonged overall survival and tumor control in an NK cell-dependent manner [102,103]. However, surprisingly, the production of IFNγ by TIM3^+^ NK92 cells, a human NK cell line, was also found enhanced upon binding to its ligand galectin-9 [104]. Interestingly, NK cells exhibited TIM3 upregulation in response to different stimuli (e.g., various cytokines such as IL-2, IL-15, IL-12, IL-18, and antibody stimulation), but the *initial* priming factor responsible for the induction of TIM3 was described to primarily influence the function of TIM3^+^ NK cells, possibly explaining some apparent discrepancies in the literature. For example, both IL-12/IL-18 and CD16-stimulation with IgG1 Fc multimers were shown to induce TIM3 expression on NK cells but solely the former led to enhanced IFNγ production [105]. In conclusion, TIM3 was able to regulate NK cell activity both positively and negatively, suggesting that other factors might differentially influence the functionality of NK cells through TIM3 induction. Clearly, detailed analysis is required to unravel these potentially opposing effects further.

### 4.4. TIGIT-CD96

TIGIT (T cell immunoglobulin and immunoreceptor tyrosine-based inhibitory motif domain) and CD96 are two inhibitory receptors that are expressed by NK cells and T cells and bind to the poliovirus receptor (PVR and CD155) and nectin (CD112), which are expressed by antigen-presenting cells and, upon cellular stress, by malignant and virus-infected cells [106,107]. These receptors are described in the same paragraph as they both bind the ligands of DNAX Accessory Molecule-1 (DNAM1), an NK cell activating receptor, thus potentially outcompeting its stimulatory effects. TIGIT^+^ NK cells have been found in PBMC, both from healthy donors and patients with cancer [74,108,109] and in the TME [74,110]. It was shown *both in vitro* and *in vivo* that TIGIT could impair NK cell activity upon binding with its ligands and that NK cell function could be restored by blocking TIGIT [106,108,110,111] (Figure 3). The role of NK cells in TIGIT blockade-based therapies was analyzed in multiple mouse models where the efficacy of TIGIT blockade was dependent on both direct NK cell activation and on NK cell-dependent secondary T cell activation. Upon NK cell depletion, CD8^+^ T cells expressed less CD107a, IFNγ, or TNFα, suggesting that NK cells support CD8^+^ T cell function and/or prevent their exhaustion. Moreover, in NK cell-deficient mice, the therapeutic effect of TIGIT blockade was abolished even in the presence of TIGIT^+^CD8^+^ T cells, suggesting that, in this setting, NK cells support CD8^+^ T cell function [110]. 

CD96^+^ NK cells were found in PB of both healthy donors and patients with cancer and in the TME [109,112]. The effects of CD96 on NK cell activity were studied ex vivo using mouse NK cells wherein IFNγ production by NK cells was impaired in the presence of a stimulating CD155-Fc antibody construct, whereas the addition of an anti-CD96 mAb or the use of CD96^−/−^ NK cells could reverse this effect (Figure 3). Moreover, CD96 had higher in vitro affinity for CD155 as compared to DNAM1/CD226, an NK cell activating receptor that recognizes the same ligands, suggesting that CD96 could hinder DNAM1-dependent NK cell activation [113]. The contribution of NK cells to the efficacy of CD96 blockade was studied in multiple mouse models, where an antagonistic anti-CD96 improved metastasis control in an NK cell and IFNγ dependent manner [107,112,114,115]. Interestingly, the effects of anti-CD96 were found to also be dependent on the presence of DNAM1, suggesting that CD96 is a critical regulator of DNAM1-dependent NK cell activation, likely through the aforementioned competition for binding to ligands [107,113,115]. Finally, in mice, the combination of anti-CD96 mAb with other immune checkpoint inhibitors (i.e., CTLA-4 and PD-1) or chemotherapy (e.g., doxorubicin and gemcitabine) demonstrated to be beneficial, suggesting a supportive role for CD96 blockade in combination with other anti-cancer therapies [107,114]. In line with the murine results, CD96^+^ healthy donor NK cells showed lower levels of activation compared to CD96^−^ NK cells. In the same study, CD96^+^ NK cells were co-cultured with a CD155 expressing cell line, and higher levels of cytotoxicity were found in the presence of a CD96 blocking mAb [112], indicating that also in humans, CD96 could play an important role in the regulation of NK cell activity. In conclusion, both TIGIT and CD96 were demonstrated to negatively regulate NK cell activity, and preventing the binding of these receptors with their ligands could benefit NK cell-mediated anti-tumor functions.

### 4.5. LAG3

Lymphocyte activation gene-3 (LAG3) is an immune checkpoint receptor that has been described to be expressed on both activated T cells and NK cells [116]. However, yet little is known about its role on NK cells. In one human study, the presence of anti-LAG3 blocking mAbs or a recombinant soluble form of LAG3 did not influence LAG3^+^ NK cell cytotoxicity against multiple tumor cell lines, suggesting that in humans, LAG3 may not have a major influence on NK cell activity [117].

In conclusion, multiple immune checkpoints contribute to the regulation of NK cell activity. PD-1, TIGIT, and CD96 can negatively affect NK cell function, and targeting these receptors with immune checkpoint inhibitors was found to enhance the anti-tumor response both in vitro and in vivo. In contrast, TIM3 was described to potentially both enhance and inhibit NK cell function, suggesting that other factors play a role in this pathway, urging the need for further studies. CTLA-4 blockade was observed to indirectly influence NK cells by reducing Treg and tumor-related immune suppression, but the potential ADCC effect by NK cells and its possible role in anti-CTLA-efficacy is still unclear. Finally, although LAG3 can be expressed by NK cells, its role on NK cell activity seems to be limited.

## 5. Chemotherapy

Experimental and clinical studies have shown that some chemotherapeutic agents may influence anti-cancer immune responses by directly modulating NK cell function [118,119,120,121]. Moreover, in response to cytotoxic agents, stressed or dying tumor cells up-regulate the expression of death receptors and different NK cell ligands on tumor cells and release DAMPs, which indirectly influence the immune response in the TME [118,119,122] (Figure 2). Below different classes of chemotherapeutic agents, according to their mechanism of action, will be discussed in relation to their effects on NK cells and the possible involvement of NK cells in their efficacy. 

### 5.1. Alkylating and Alkylating-Like Agents 

Alkylating agents are chemotherapeutic drugs characterized by the ability to transfer alkyl-group to DNA bases leading to DNA damage and cell cycle arrest [123]. These compounds were shown to also directly affect NK cell function. In vitro, a reduction of NK cell cytotoxicity was reported upon exposure to chlorambucil [124]. Similarly, ifosfamide decreased in vitro NK cell activity in a dose-dependent manner through the reduction of intracellular glutathione [125,126,127]. Interestingly, cyclophosphamide indirectly enhanced ex vivo NK cell activity when administered in low doses in patients with end-stage cancer by inducing a selective depletion of Treg. However, this effect was lost when the dose of cyclophosphamide was increased, causing pan-lymphopenia [128]. 

Platinum drugs are chemotherapeutic agents that can form covalent bonds between the DNA and the platinum component, leading to DNA damage. These compounds are also known as alkylating-like agents due to the similarity in the mechanism of action [123]. Oxaliplatin and carboplatin had minimal impact on in vitro NK cell activity against the cell line K562 [124], whereas the exposure to single-dose oxaliplatin was observed to upregulate multiple NK cell-activating ligands (NKG2D, DNAM1, and TRAIL ligands) on ovarian cancer cells leading to increased NK cell-mediated lysis [129]. In contrast, no changes in the expression of NK cell ligands were found when multiple neuroblastoma and breast cancer cell lines were exposed to single-dose cisplatin [129,130]. It is noticeable that oxaliplatin, but not cisplatin, was shown to induce immunologic cell death, possibly explaining such apparent discrepancies [131]. Moreover, different responses to cisplatin have been described using other tumor cell lines, suggesting that the effects might also be dependent on the tumor cell type. For example, it was reported that single and repeated *in vitro* exposure of non-small cell lung cancer (NSCLC) cell lines to cisplatin lead to the upregulation of MICA/B through the regulation of ataxia-telangiectasia mutated (ATM), ataxia-telangiectasia, and Rad3-related (ATR) signaling pathways, as well as other NKG2D-L (ULBP1, ULBP2/5/6, ULBP3, and ULPB4), resulting in increased NK cell activity [132,133]. Similarly, HCC cell lines exhibited higher levels of ULBP2 upon exposure to low doses of cisplatin, which was also observed to enhance tumor control in HCC-bearing mice when combined with allogeneic NK cells [134]. In contrast, in patients with NSCLC, treated with more than two cycles of cisplatin-based chemotherapy followed by surgery, MICA/B and ULBP 2/3/4 were shown to be downregulated in 5/10 patients after treatment [133]. A possible explanation for this discrepancy is that the *in vivo* upregulation of these ligands by a subpopulation of tumor cells might have induced a selective NK cell response resulting in a general reduction in the expression. Cisplatin was also found to enhance the *in vitro* expression of B7-H6, a ligand of the activating NKp30 NK cell receptor, on tumor cells, making them more sensitive to NK cell lysis [135]. Finally, both alkylating and platinum agents were shown to induce the release of DAMPs, such as the high-mobility group box 1 protein (HMGB1), which can activate the innate immune system [131,136], and ATP, which, as mentioned, can be enzymatically converted to ADO leading to immune suppression [137]. 

### 5.2. Microtubule Targeting Agents

Microtubule targeting agents (MTAs) are a group of chemical compounds with the ability to interfere with the function of microtubules leading to cell death [138]. *In vitro*, multiple MTAs were described to substantially reduce NK cell cytotoxicity in a dose-dependent manner [124]. Similarly, patients with NSCLC treated with paclitaxel exhibited lower levels of *ex vivo* NK cell cytotoxicity [139]. These findings can be explained considering that NK cell activity was shown to be dependent on microtubule and microfilament integrity [140]. Moreover, a decrease in pbNK cells occurred in patients with various advanced cancers treated with docetaxel. Interestingly, this decrease was not shown in patients treated with paclitaxel alone [139,140] or in combination with carboplatin [141]. The reasons for this discrepancy are currently unclear and, as reductions in pbNK frequency were also observed following docetaxel treatment, this effect is probably not related to the chemotherapeutic class. MTAs were described to also influence NK cell activity indirectly. *In vitro*, cytochalasin D, nocodazole, and docetaxel induced the expression of several NKG2D and DNAM1 ligands on tumor cells leading to increased sensitivity to NK cell-mediated killing [142,143]. Interestingly, docetaxel enhanced NKG2D expression on NK cells in patients with breast cancer, suggesting a higher lytic ability [143].

### 5.3. Antimetabolites

Antimetabolites are small molecules related to nucleotide metabolites with the ability to interfere with DNA replication [123,144]. Multiple antimetabolite agents (e.g., methotrexate and fluorouracil) were described to have minimal or no effects on *in vitro* NK cell function except for the purine antagonist cladribine [124]. However, some antimetabolites indirectly affect NK cell function. Melphalan triggered the DNA damage response pathway of various multiple myeloma (MM) cell lines, which stimulated the *in vitro* up-regulation of MICA/B and PVR through the ATM-ATR pathway [145,146]. Also, 5- fluorouracil induced the *in vitro* expression of B7-H6 on tumor cells, making them more susceptible to NK cell killing [135]. 

### 5.4. Anthracyclines

Anthracyclines are a group of chemotherapeutic drugs extracted from Streptomyces species plural that function through multiple mechanisms of action, including interacting with the enzyme topoisomerase-II, interfering with DNA replication, and inducing the release of reactive oxygen species [147]. Daunorubicin, epirubicin, and doxorubicin were shown to have minimal effects on *in vitro* NK cell activity against K562 [124]. On the other hand, similarly to the antimetabolite melphalan, doxorubicin enhanced the *in vitro* expression of MICA/B and PVR on various MM cell lines through the ATM-ATR signaling [145,146]. In line with this, epirubicin induced higher levels of MICA/B, ULBP1/2, and Fas on breast cancer cell lines resulting in higher *in vitro* NK cell-mediated oncolysis [148]. In contrast, no changes in MICA/B were found on other tumor cell lines (renal cell carcinoma (RCC), melanoma, and bladder cancer) treated with doxorubicin. In this setting, higher levels of TRAIL and increased sensitivity to NK cell-mediated lysis were detected [149], suggesting differences in the NK cell response based on tumor type. 

### 5.5. Other Anti-Cancer Agents 

Histone deacetylases inhibitors (HDACi) are epigenetic regulators used for the treatment of hematological malignancies [150]. These agents demonstrated to inhibit NK cell activity. The *in vitro* exposure of NK cells to the HDACi valproic acid (VPA) and suberoylanilide hydroxamic acid decreased NK cell degranulation and cytotoxicity [151,152]. Moreover, in patients with cutaneous T-cell lymphoma treated with romidepsin, lower levels of K562-induced NK cell degranulation were observed compared to pretreatment levels [153]. HDACi were also found to modulate the expression of various ligands on the surface of tumor cells. In particular, HDACi upregulated NKG2D-L, such as MICA/B and ULBP1, on multiple tumor cell lines, both from hematological and solid tumors and on patient-derived acute myeloid leukemia (AML) cells, leading to increased sensitivity to NK cell-mediated cytotoxicity [154,155,156,157]. Similarly, VPA enhanced NK92-mediated tumor control in a pancreatic-mouse model by upregulating MICA/B [155]. HDACi were also shown to downregulate B7-H6 leading to the reduction of NK cell degranulation also against cell lines upregulating NKG2D-L in response to HDCAi, suggesting that this might limit NK cell response efficacy induced by HDACi [158]. Finally, various HDACi were described to induce the expression of TRAIL receptors on various malignant cell types [159,160,161].

Proteasome inhibitors are anti-cancer agents targeting the ubiquitin-proteasome pathway [162]. Bortezomib, a proteasome inhibitor approved for relapsed and refractory MM [162], was found to negatively impact NK cell activity *in vitro* in a dose-dependent manner [124]. Interestingly, at a low dose, bortezomib enhanced the expression of MICA/B on hepatocellular lines without negatively affecting NK cell activity [163]. Similar results were observed with MM cell lines and patient-derived malignant plasma cells in which higher levels of PVR and Nectin-2 upon *in vitro* exposure to low-dose bortezomib were found [145,164]. However, no changes in the expression of NKG2D-L have been described on other MM and various RCC cell lines cultured with low-dose bortezomib [165,166], suggesting differences in response to bortezomib. Finally, bortezomib enhanced the expression of DR5, a TRAIL ligand, on various tumor cells, increasing NK cell-mediated cytotoxicity [166,167,168]. 

### 5.6. Combination Therapies

Apart from single class effects, chemotherapeutic agents are often given in combination to maximize the anti-tumor effects. The effects of adjunctive chemotherapeutic treatments on pbNK cells frequency have been analyzed in various patient groups showing reductions in pbNK cell frequency after chemotherapy in patients with NSCLC (cisplatin/nedaplatin and pemetrexed), triple-negative breast cancer (epirubicin and cyclophosphamide), colorectal cancer (5-fluorouracil, oxaliplatin, and leucovorin) [169,170,171]. Combination therapies were also found to impact NK cell activity. Two studies analyzing the effects of chemotherapy (5-fluorouracil, cyclophosphamide combined with adriamycin or methotrexate) in patients with breast cancer reported a reduction in *ex vivo* NK cell-mediated cytotoxicity compared to pretreatment [172,173]. Similarly, a decline in NK cell activity was observed in patients with various stages of breast cancer treated with different chemotherapy regimens [174]. In contrast, a study analyzing patients with advanced ovarian cancer, treated with paclitaxel and carboplatin, found no change in NK cell activity after chemotherapy [141]. Interestingly, studies comparing different chemotherapy regimens in patients with cancer reported that the effects of the agents on NK cell frequency and activity differed based on both disease type and intensity of the therapy [174,175], possibly explaining the apparently conflicting reports on NK cell responses.

In conclusion, despite differences between the various chemotherapeutic agents, the exposure of NK cells to chemotherapy overall seems to reduce their activity while it increases the sensitivity of tumor cells to NK cell killing, suggesting that strategies to maintain NK cell activity during chemotherapy might be beneficial. 

## 6. Protein Kinase Inhibitors 

Protein kinase inhibitors are a group of agents that exert their function by blocking the activity of one or more protein kinases [176,177], enzymes that play a vital role in the regulation of important cellular pathways [178,179]. The dysregulation of protein kinases leads to multiple pathological conditions, including cancer [180,181,182]. Some protein kinase inhibitors have both a direct and indirect effect on NK cell activity (Figure 2). 

### 6.1. Src-Kinase Inhibitors

Multiple protein tyrosine kinases of the Src-family were found to be involved in NK cell activity [183]. Dasatinib, a second-generation broad Src-kinase inhibitor used for the treatment of chronic myeloid leukemia (CML), reduced in vitro NK cell functions in a dose-dependent manner [184]. In contrast, NK cells isolated from patients with leukemia treated with dasatinib showed higher levels of ex vivo degranulation and cytotoxicity compared to either pretreatment measurement or untreated controls, suggesting differential effects of in vitro versus in vivo exposure [185,186]. Moreover, higher levels of ex vivo NK cytotoxicity were found in the patients with a complete cytogenetic response [186], which might be related to the eradication of the tumor and, consequently, of the tumor-related immune suppression. In contrast, findings on imatinib, another broad Src-kinase inhibitor approved for the treatment of various cancer types, are less clear. While one study described higher levels of *ex vivo* NK cell degranulation after 3 months of therapy [187], others found no changes in NK cell activity compared to either baseline or untreated controls both immediately after administration (2 h) and after long term therapy (median: 936 days; range: 28–2448) [185,186]. This discrepancy could, at least partially, be explained by the different time points at which NK cell activity was analyzed. Interestingly, in patients with CML treated for at least 3 years with imatinib and with a sustained molecular response, higher levels of effector NK cells at imatinib discontinuation correlated to better relapse-free survival, suggesting that NK cells have a role in tumor control after therapy discontinuation [188,189]. 

### 6.2. BRAF Inhibitors

As a part of the MAPK pathway, which regulates cell proliferation, survival, and metastasis [190], B-rapidly accelerated fibrosarcoma (BRAF)/MAPK has a significant role in cancer development [191]. Currently, there are three BRAF inhibitors (BRAFi) approved for clinical use in the treatment of advanced melanoma: vemurafenib, dabrafenib, and encorafenib [192,193]. The *in vitro* exposure to vemurafenib was shown to enhance ERK1/2 phosphorylation, CD69 expression and induce higher levels of IL-2 dependent IFNγ production in NK cells suggesting that BRAFi can directly enhance NK cell function. Moreover, in a mouse model, NK cells played a critical role in the antimetastatic function of vemurafenib in a perforin-dependent manner, which was enhanced by the combination with IL-2 [194]. In contrast, vemurafenib indirectly hampered NK cell activity by modulating the expression of various NK cell ligands on the surface of tumor cells. In particular, vemurafenib downregulated expression of the activating MICA and ULBP2 ligands while it upregulated expression of the inhibitory HLA-E and HLA-ABC receptors [195,196]. Interestingly, in melanoma cell lines, the downregulation of MICA and ULBP2/5/6 induced by vemurafenib was counterbalanced by the addition of HDACi, potent NKG2D ligand inducers, to the *in vitro* culture, leading to enhanced NK cell activity [196]. This suggests that the combination with HDACi could increase the efficacy of BRAFi in an NK cell-dependent manner. Finally, different melanoma cell lines with acquired resistance to vemurafenib and dabrafenib were described to modulate the expression of various NK cell ligands (e.g., MICA/B and HLA-ABC), resulting in higher sensitivity to NK cell lysis compared to the parental cell lines, suggesting patients gaining such resistance might benefit from strategies to enhance NK cell activity [197,198]. 

### 6.3. GSK-3β Inhibitors

Glycogen synthase kinase-3β (GSK-3β) is a Serine/Threonine kinase that plays a pivotal role in many cellular processes. Although multiple GSK-3β inhibitors are currently under preclinical and clinical evaluation, no compound has yet been approved for cancer treatment [199]. In NK cells, active GSK-3β has been associated with functional impairment and in line treatment with GSK-3β inhibitors increased in vitro cytotoxicity mediated by NK cells derived from both healthy donors and patients with cancer [200,201]. GSK-3β was shown to be inhibited via ERK or AKT signaling upon binding of NKG2D to its ligands, leading to activation of NK cells, confirming the negative regulatory role of GSK-3β in NK cell function [202]. Moreover, in MM cell lines treated with a GSK-3β inhibitor, higher expression levels of MICA were found, which was further augmented in combination with chemotherapeutics, and significantly improved NK cell cytotoxic activity through NKG2D recognition, suggesting that also indirect effects of GSK-3beta therapy can affect NK cell activity [203].

### 6.4. Other Protein Kinase Inhibitors

Sunitinib and sorafenib are two multi-target small-molecule tyrosine kinase inhibitors (TKI) used for the treatment of multiple cancers [192]. In vitro, exposure of NK cells to pharmacological levels of sorafenib, but not sunitinib, suppressed NK cell cytotoxicity and IFNγ production by reducing the levels of IL-2-induced ERK1/2 phosphorylation [204]. Interestingly, NK cell activity and ERK1/2 phosphorylation were enhanced when NK cells were exposed to a lower dose of sorafenib, indicating a dose-dependent effect [205]. Sunitinib and sorafenib indirectly enhanced *in vitro* NK cell activity against tumor cell lines by inducing the expression of NKG2D ligands on tumor cells through the noncanonical NF-κβ signaling pathway [206,207].

Janus kinase (JAK) and signal transducer and activator of transcription proteins (STATs) are part of a signaling pathway that mediates cellular response to cytokines and growth factors. This pathway was found to be critical for NK cell development and activation [208]. Ruxolitinib, the first JAK 1/2/(3) inhibitor approved for clinical use, was shown to directly inhibit NK cell function and maturation [209]. In contrast, other JAK inhibitors were found to indirectly enhance NK cell activity *in vitro* by impeding downregulation of NKG2D ligands and inducing upregulation of PD-L1 in NSCLC and PC cell lines, by IFNγ and IL-6 respectively, leading to enhanced NK cell-mediated killing [133,210]. It is currently unclear which of these contrasting effects prevails in vivo, thus determining the NK cell response. 

Casitas B-lymphoma (Cbl)-b is an E3 protein ubiquitin-ligase expressed by all leukocytes that negatively regulate immune activation. Multiple drugs targeting one or more members of this family have currently been approved for clinical use for various cancer types (e.g., NSCLC, CML, and AML) [211]. On NK cells, Cbl-b was shown to target members of the TAM receptor family (Tyro3, Axl, and Mer), interfering with NK cell function [212]. In agreement with these findings, the administration of an experimental TAM inhibitor in multiple mouse tumor models led to the release of IFNγ and NK cell-dependent tumor control [213]. 

In conclusion, multiple protein kinases directly reduce NK cell activity, and the inhibition of these pathways through protein kinases inhibitors is mostly beneficial except for the JAK kinase inhibitors, which were shown to negatively impact NK cell functions. Moreover, several protein kinase inhibitors were observed to modulate the expression of various NK cell ligands on the surface of tumor cells, thus indirectly regulating NK cell activity.

## 7. Oncolytic Viruses 

**Figure 4:**
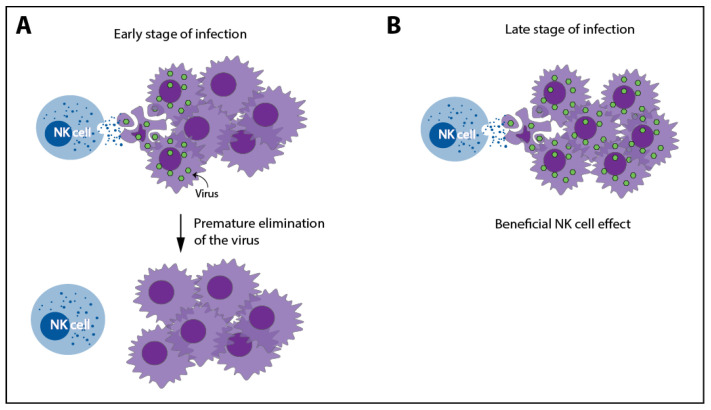
A dual role of NK cells in oncolytic virus therapies. Due to their ability to recognize and kill virus-infected cells, NK cells can cause a premature elimination of the virus, limiting its efficacy (**A**). However, thanks to this ability, NK cells can also enhance the clinical response by eliminating tumor cells in later stages of oncolytic virus (OV) therapy (**B**).

Oncolytic virus (OV) immunotherapy is a therapeutic approach that exploits native or genetically engineered viruses to selectively replicate in tumor cells leading to cell lysis and immune activation through the release of neoantigens, pathogen-associated molecular patterns, DAMPs, and various cytokines (e.g., type-I IFN, TNFα, IFNγ, and IL-12) [214,215]. Currently, multiple oncolytic viruses are under clinical evaluation. In 2005, the use of Oncorine, an engineered adenovirus, was approved in China for the treatment of nasopharyngeal carcinoma. Moreover, the FDA approved the use of Talimogene laherparepvec, an engineered oncolytic herpes simplex virus (oHSV), for the treatment of advanced melanoma [216]. Although to our knowledge, the role of NK cells in approved OV therapies has not been analyzed, in multiple experimental OV therapies, NK cells were described to play a dual role, both enhancing and limiting their therapeutic efficacy (Figure 4).

In support of the former, it was described that OVs enhance specific NK cell anti-tumor activity against various tumor cell lines. This increase was shown to be related to higher expression of NCR ligands or the downregulation of MHC-I [217,218,219,220,221]. In mice, multiple OVs induced mobilization, tumor recruitment, and activation of NK cells [222]. Moreover, in tumor bearing-mice, the treatment with an oHSV or an oncolytic Vesicular Stomatitis Virus (oVSV) was hampered when NK cells, or CD8^+^ T cells, were depleted [223,224]. Interestingly, the preoperative administration of oncolytic parapoxvirus ovis could counteract surgery-induced suppression of NK cells, leading to higher metastatic control in tumor-bearing mice [225]. 

However, NK cells can also act as antagonists of OV therapy thanks to their ability to recognize virus-infected cells, which can often lead to the premature elimination of virus-infected tumor cell, thus interfering in the further viral spread and sequential waves of oncolysis, thus reducing the actual efficacy of this therapy [214]. In HCC-bearing rats treated with oVSV, NK cell depletion or strategies to hamper NK cell tumor-migration were found to increase OV efficacy [226]. Similarly, a novel oHSV, encoding E-cadherin, an adhesion molecule, and a ligand for killer cell lectin-like receptor G1, an inhibitory receptor expressed by NK and T cells, increased survival in glioblastoma-bearing mice by facilitating the cell-to-cell infection and preventing NK cell-mediated killing [227]. Moreover, in other glioblastoma-mouse models, it was shown that NK cell depletion or the combination with HDACi, which impaired NK cell anti-viral activity, enhanced the efficacy of oHSV [228,229]. In conclusion, NK cells exert both agonist and antagonistic effects in OV-based therapies. Interestingly, the ability of these therapies to enhance NK cell function might play a positive role when combined, with the appropriate timing, with other anti-cancer therapies that modulate NK cell activity and the TME. In line with this hypothesis, a study analyzing the optimal NK cell response dynamics during OV-bortezomib therapy showed that a temporary NK cell-depletion before virus therapy followed by NK cell adjuvant therapy administered after OV-bortezomib therapy might maximize the therapeutic benefits [230].

## 8. Conclusions

In this review, we have discussed how different anti-cancer therapies affect and are affected by NK cells. All considered treatments were found to impact NK cell function, while less is known on the contribution of NK cells to the efficacy of these treatments. Conventional anti-cancer therapies, such as radiotherapy and chemotherapy, which induce non-specific cell damage, were demonstrated to impact the viability and the function of both NK and tumor cells. The latter, upon genotoxic stress, was described to enhance the expression of NK cell ligands and to release DAMPs leading to both enhanced and impaired NK activity. Immune checkpoint and protein kinases inhibitors were mostly found to release NK cell activity by targeting specific receptors or pathways involved in dampening NK cell function. Moreover, protein kinase inhibitors also indirectly affected NK cells by modulating the expression of NK cell ligands on the surface of tumor cells. Results from animal studies suggest that NK cells contribute to the efficacy of some checkpoint inhibitors (i.e., PD-1-PD-L1, TIM3, TIGIT, and CD96) and protein kinase inhibitors (i.e., BRAFi). The findings on local ablation therapies are less clear due to the limited amount of available data and discrepancies in results. Therefore, no firm conclusions can be drawn. However, NK cell dynamics were correlated with efficacy in patients treated with MWA and RFA, suggesting that NK cells play a role in response to these treatments. Finally, NK cells were described to play a dual role in OV therapies, being activated by the therapy, on the one hand, thus contributing to tumor kill, but recognizing virus-infected cells, leading to the premature clearance of the OV, on the other hand. In mouse studies, NK cell depletion or strategies to hamper their function were both found to abolish and enhance the efficacy of OVs. In this setting, the timing of NK cell activation might be relevant in determining a more positive or negative effect. A better understanding of NK cell function in anti-cancer therapies could lead to a rationale for combining these treatments with strategies to modulate NK cell activity and thereby increase therapeutic efficacy. For example, bispecific antibodies or Fc-optimized mAb can represent useful strategies to enhance NK cell recognition and activation to specific targets [6]. Similarly, OVs might also induce specific NK cell effector function when combined, at the right time, with therapies that impair NK cell activity. NK cell allogeneic transfer could also represent a useful approach to increase NK cell numbers and tumor recognition, particularly in the setting of KIR or HLA mismatch [6]. The development of chimeric antigen receptor (CAR)-NK cells might further enhance the tumor specificity of allogeneic NK cells leading to promising future combination therapies. In conclusion, NK cells play a pivotal role against cancer and multiple anti-cancer therapies through various mechanisms have an impact on their function. The understanding of the underlying mechanisms opens opportunities to enhance NK cell activity and has the potential to increase the anti-cancer efficacy of these therapies.

## Data Availability

No new data were created or analyzed in this study. Data sharing is not applicable to this article.

## Supplementary Materials

The following are available online at https://www.mdpi.com/2072-6694/13/4/711/s1, Table S1: Clinical evaluations and studies included.
