# Supplementary material for: Natural Killer Cells and Anti-Cancer Therapies: Reciprocal Effects on Immune Function and Therapeutic Response
Source: Cancers (Basel). 2021 Feb 9;13(4):711. doi: 10.3390/cancers13040711 (PMC7916216; doi:10.3390/cancers13040711)
Supplement: Supplementary file 1 [file cancers-13-00711-s001.pdf]

Supplementary Table 1: Clinical evaluations and studies included.

Reference	Therapy	Patient details	Effects on/of NK cells
Eric et al. 2009 **[19]**	RT	39 patients with cervical cancer treated with external irradiation and concomitant intracavitary brachytherapy. (Total dose: external RT 64 Gy; brachytherapy 35 Gy).	Decreases in the absolute number of pbNK cells compared to pre-treatment.
Clave et al. 1995 **[21]**	RT	30 patients with various malignant hematological disorders treated with total body irradiation as part of their conditioning regimen pre-transplantation. (Total dose 12 Gy).	Decreases in the absolute number of pbNK cells compared to pre-treatment. More important reductions in the $CD56^{+}CD16^{-}$ subset.
Louagie et al. 1999 **[22]**	RT	8 patients with carcinoma of the cervix or endometrium treated with external beam RT of the pelvis. (Total dose 46-50 Gy.	Reductions in the absolute number of pbNK cells compared to pre-treatment. Limited during the first weeks of therapy and higher afterwards.
Mozaffari et al. 2007 **[23]**	RT	41 patients with breast cancer treated with RT or RT+CT were compared to 9 pretreatment patients with breast cancer. (Total dose: 50 Gy delivered to the breast).	Reductions in both absolute pbNK cell numbers and *ex vivo* cytotoxicity in patients treated with RT and RT+CT compared to pre-treatment controls. Stronger decrease in RT+CT patients.
Nakayama et al. 1995 **[24]**	RT	15 patients with lung cancer undergoing RT including bilateral mediastinal lymph nodes. (Total dose 40-50 Gy).	Decrease in both the absolute number and the percentage of pbNK cells compared to pre-treatment.
Belka et al. 1999 **[25]**	RT	10 patients with seminoma testis treated with RT. (total dose 26 Gy delivered to paraaortal lymph nodes).	Decline in the absolute number of pbNK cells compared to pre-treatment, which normalized after 6 weeks.
Domouchtsidou et al. 2018 **[26]**	RT	25 patients with various inoperable hepatic malignancies treated with selective internal radiotherapy (^{90}Y radioactive glass microspheres)	Decrease in the absolute number of pbNK compared to pre-treatment.
Yamaue et al. 1992 **[27]**	RT	11 patients with advanced	Reduction in *ex vivo* pbNK

		cancer treated with RT (total dose: 25-35 Gy)	cell cytotoxic activity compared to pre-treatment.
Blomgren et al. 1980 **[29]**	RT	24 patients with breast cancer, 6 patients with prostate cancer, and 7 patients with carcinoma of the urinary bladder treated with local radiotherapy (total dose 45-64.5 Gy)	Reduction in *ex vivo* pbNK cell cytotoxic activity compared to pre-treatment.
Urosevic et al. 2005 **[37]**	RT	Tissue from 38 primary (untreated) and 14 relapsed, after RT, basal cell carcinomas.	Lower levels of HLA-G were found in the relapsed tumors.
Ames et al. 2015 **[39]**	RT	Tissue from 12 patients with pancreatic ductal adenocarcinoma, Ewing sarcoma, and leiomyosarcoma treated with neoadjuvant RT.	Enrichment of cancer stem cells and NKG2D ligands (MICA/B) compared to pre-treatment biopsies and untreated patients with sarcoma.
Matuszewski et al. 2011 **[55]**	RFA	6 patients with renal cell cancer.	No significant changes in the number of pbNK cells compared to pre-treatment.
Zerbini et al. 2010 **[56]**	RFA	37 patients with HCC.	Increase in the absolute number of pbNK cells compared to pre-treatment. Mostly due to increase in $CD56^{dim}$ NK cells and accompanied by increasedexpression of various NK cell-activating receptors (NKG2D, CD16, NKp30, NKp46), reduced expression of the inhibitory receptor NKG2A and higher *in vitro* NK cell cytotoxicity, ADCC, and IFNγ production compared to baseline. Increased IFNγ-levelsand NK cell cytotoxicity 4 weeks after RFA associated with longer disease-free survival.
Guan et al. 2013 **[57]**	RFA	9 patients with HCC.	Increased pbNK cell levels compared to pre-treatment.
Rochigneux et al. 2019 **[58]**	RFA	80 patients with HCC.	Reduction of $NKp30^{+}$ pbNK cells at day 1 after treatment which normalized after 1

			month. Higher frequency of NKp30$^+$ pbNK cells 1 day after RFA correlated to lower tumor recurrence whereas a delayed increase of total NK cells and higher percentages of CD56^{bright} NK cells at 1 month after RFA were associated with more tumor recurrence.
Yu et al. 2020 **[60]**	MWA	14 patients with breast cancer.	Increased percentage of NK cells at 1 week with increased proportion of CD16$^+$ CD56$^+$ NK cells. Higher levels of NKp46$^+$ CD3$^-$ CD56$^+$ cells; No increase in NKG2A$^+$ CD3$^-$ CD56$^+$cells.
Dong et al. 2003 **[61]**	MWA	89 nodules from 82 patients with HCC.	Stronger NK cell infiltration in treated and untreated lesions compared to pre-treatment. Stronger NK cell infiltration correlated to lower recurrence rate.
Zhang et al. 2017 **[62]**	MWA	45 patients with HCC.	No significant changes in pbNK cell frequency. compared to pre-treatment.
Szmigielski et al. 1991 **[63]**	MWA	15 patients with advanced adenocarcinoma of the prostate + 15 patients with severely symptomatic benign prostatic hyperplasia treated with transrectal microwave hyperthermia	Higher levels of *ex vivo* pbNK cell cytotoxicity after treatment in patients with cancer. More pronounced increase in patients with CR or PR.
F. Wu et al. 2004 **[65]**	HIFU	16 patients with solid malignancies.	No significant differences in pbNK cell frequency and cytotoxicity compared to pre-treatment.
Wang et al. 2013 **[66]**	HIFU	60 patients with uterine fibroids.	No significant differences in pbNK cell frequency compared to pre-treatment.
Ma, Liu, and Yu 2019 **[67]**	HIFU	96 patients with primary liver cancer.	Higher levels of pbNK cells compared to pre-treatment were measured with enzyme-linked immunosorbent assay.
Lu et al. 2009 **[68]**	HIFU	48 patients with breast cancer undergoing mastectomy of which 23	HIFU treated patients had higher intratumoral levels of CD57+ cells compared to

		were treated with HIFU prior surgery. Tissue analyzed after surgery.	untreated controls.
Sanseviero et al. 2020 **[91]**	αCTLA-4 CI	RNA-seq data from patients with malignant melanoma treated with ipilimumab.	Higher levels of CD56 cells in patients that benefit from the therapy.
Tallerico et al. 2017 **[92]**	αCTLA-4 CI	67 melanoma patients treated with ipilimumab.	Increased frequency of $CD56^{dim}$ $CD16^{+}$ pbNK cells correlated with survival.
Sottile et al. 2019 **[93]**	αCTLA-4 CI	27 malignant mesothelioma patients treated with tremelimumab.	A perturbation of the $CD56^{dim}/CD56^{bright}$ ratio was found compared to healthy donors which normalized after treatment. Overall survival correlated with high $DNAM1^{+}$ $CD56^{dim}$ pbNK cells and increased levels of NKp46 after treatment.
Tietze et al. 2017 **[94]**	αCTLA-4 CI	32 patients with metastatic melanoma treated with ipilimumab.	Increase in the proportion of $CD56^{dim}$ NK cells in patients with low baseline levels thereof after treatment.
Okita et al. 2019 **[135]**	CT	10 patients with NSCLC treated with neoadjuvant platinum-based chemotherapy. Tissue was collected before and after surgery.	Decrease in levels of MICA/B in 2 patients<br>Reduced expression of ULBP-2/5/6 in 4 patients
Sako et al. 2004 **[141]**	CT	10 patients with NSCLC treated with paclitaxel.	Decrease in *ex vivo* pbNK cell activity at 1 and 8 days after treatment; no difference in pbNK cell frequency.
Tong et al. 2000 **[142]**	CT	10 patients with various advanced cancers treated with docetaxel (N=5) or paclitaxel (N=5).	Decrease in pbNK cell frequency at 1 and 4 weeks after docetaxel but not after paclitaxel compared to pre-treatment.
X. Wu et al. 2010 **[143]**	CT	13 patients with advanced ovarian cancer treated with carboplatin plus paclitaxel.	No significant difference in NK cell frequency and *ex vivo* cytotoxicity compared to pre-treatment.
di Modica et al. 2016 **[145]**	CT	8 patients with breast cancer treated with 3-4 cycles adriamycin plus docetaxel.	Significantly higher expression of NKG2D on NK cells after CT compared to pre-treatment.
Kelly-Sell et al. 2012 **[155]**	CT	8 patients with cutaneous T-cell lymphomas treated with romidepsin.	Decrease in *ex vivo* pbNK cell degranulation compared to pre-treatment levels.

Massa et al. 2020 **[172]**	CT	56 patients with triple-negative breast cancer treated with nanoparticle albumin-bound paclitaxel followed by epirubicin and cyclophosphamide (EC).	Decrease in the absolute number of pbNK cells after EC compared to pre-treatment and post paclitaxel levels.
Aldarouish et al. 2019 **[173]**	CT	50 patients with NSCLC treated with cisplatin/nedaplatin and pemetrexed.	Decrease in the absolute number and percentage of pbNK cells after CT.
Shinko et al. 2019 **[174]**	CT	10 patients with colorectal cancer treated with 5-FU, oxaliplatin and leucovorin.	Decrease in the absolute number of pbNK cells after CT. Decrease observed in various NK cell subpopulations: $CD56^{dim}CD16^{+}$, $CD56^{dim}CD16^{-}$, $CD56^{bright}$.
Beitsch et al. 1994 **[175]**	CT	10 patients with breast cancer treated with 5-fluorouracil, cyclophosphamide, adriamycin.	Decrease in *ex vivo* NK cell cytotoxicity compared to pre-treatment.
Sewell et al. 1993 **[176]**	CT	16 patients with breast cancer treated with 5-fluorouracil, cyclophosphamide, methotrexate.	Decrease in *ex vivo* NK cell cytotoxicity compared to pre-treatment.
Brenner and Margolese 1991 **[177]**	CT	Patients with breast cancer receiving no treatment, various CT combinations and/or endocrine therapy.	CT resulted in a decline in *ex vivo* NK cell function. More pronounced in more advanced patients.
Ogura et al. 2016 **[178]**	CT	25 patients with adult T-cell leukemia–lymphoma (N=14) or peripheral T-cell lymphoma (N=11) receiving various CT combinations.	Decrease in NK cell frequency and *ex vivo* function dependent on disease type and intensity of CT.
Mustjoki et al. 2013 **[188]**	PKI	55 patients with leukemia treated with either dasatinib (N=37), imatinib (N=8), nilotinib (N=8) or bosutinib (N=2).	No differences in *ex vivo* NK cell degranulation and cytotoxicity after imatinib or nilotinib whereas this was increased after dasatinib compared to pre-treatment levels and healthy controls.
Hayashi et al. 2012 **[189]**	PKI	63 patients with chronic myeloid leukemia treated with either imatinib (N=36), nilotinib (N=9), dasatinib (N=18).	Increased NK cell cytotoxicity after dasatinib but not after imatinib and nilotinib. In the dasatinib group, the levels of NK cell cytotoxicity tended

			to be higher in patients with complete cytogenetic response.
Kreutzman et al. 2019 **[190]**	PKI	33 patients with chronic myeloid leukemia treated with imatinib (N=20) or bosutinib (N=13).	Significant increase in the proportion of CD56+ CD16+ NK cells and NK cell degranulation after imatinib but not bosutinib.
Ilander et al. 2017 **[191]**	PKI	100 patients with chronic myeloid leukemia treated with imatinib.	The proportion of NK cells at 1 month after discontinuation was associated with molecular relapse-free survival.
Rea et al. 2017 **[192]**	PKI	51 patients with chronic myeloid leukemia treated with imatinib.	Higher absolute number of pbNK cells ($CD56^{+}CD3^{-}$) and $CD56^{dim}$ pbNK cells at the time of imatinib discontinuation was associated with higher relapse-free survival.

pbNK: peripheral blood Natural Killer. RT: radiotherapy. RFA: radiofrequency ablation therapy. MWA: microwave ablation therapy. CI: checkpoint inhibitor therapy. CT: chemotherapy. PKI: protein kinase inhibitors. HCC: hepatocellular carcinoma. CR: complete response. PR: partial response. NSCLC: non-small-cell lung cancer. 5-FU: 5-fluorouracil.
